# Combining weakly and strongly supervised learning improves strong supervision in Gleason pattern classification

**DOI:** 10.1186/s12880-021-00609-0

**Published:** 2021-05-08

**Authors:** Sebastian Otálora, Niccolò Marini, Henning Müller, Manfredo Atzori

**Affiliations:** 1 HES-SO Valais, Technopôle 3, 3960 Sierre, Switzerland; 2 Computer Science Centre (CUI), University of Geneva, Route de Drize 7, Battelle A, Carouge, Switzerland; 3 Faculty of Medicine, University of Geneva, 1 rue Michel-Servet, 1211 Geneva, Switzerland; 4 Department of Neuroscience, University of Padova, via Belzoni 160, 35121 Padova, Italy

**Keywords:** Computational pathology, Weak supervision, Transfer learning, Prostate cancer, Deep learning

## Abstract

**Background:**

One challenge to train deep convolutional neural network (CNNs) models with whole slide images (WSIs) is providing the required large number of costly, manually annotated image regions. Strategies to alleviate the scarcity of annotated data include: using transfer learning, data augmentation and training the models with less expensive image-level annotations (weakly-supervised learning). However, it is not clear how to combine the use of transfer learning in a CNN model when different data sources are available for training or how to leverage from the combination of large amounts of weakly annotated images with a set of local region annotations. This paper aims to evaluate CNN training strategies based on transfer learning to leverage the combination of weak and strong annotations in heterogeneous data sources. The trade-off between classification performance and annotation effort is explored by evaluating a CNN that learns from strong labels (region annotations) and is later fine-tuned on a dataset with less expensive weak (image-level) labels.

**Results:**

As expected, the model performance on strongly annotated data steadily increases as the percentage of strong annotations that are used increases, reaching a performance comparable to pathologists ($\kappa = 0.691 \pm 0.02$). Nevertheless, the performance sharply decreases when applied for the WSI classification scenario with $\kappa = 0.307 \pm 0.133$. Moreover, it only provides a lower performance regardless of the number of annotations used. The model performance increases when fine-tuning the model for the task of Gleason scoring with the weak WSI labels $\kappa = 0.528 \pm 0.05$.

**Conclusion:**

Combining weak and strong supervision improves strong supervision in classification of Gleason patterns using tissue microarrays (TMA) and WSI regions. Our results contribute very good strategies for training CNN models combining few annotated data and heterogeneous data sources. The performance increases in the controlled TMA scenario with the number of annotations used to train the model. Nevertheless, the performance is hindered when the trained TMA model is applied directly to the more challenging WSI classification problem. This demonstrates that a good pre-trained model for prostate cancer TMA image classification may lead to the best downstream model if fine-tuned on the WSI target dataset. We have made available the source code repository for reproducing the experiments in the paper: https://github.com/ilmaro8/Digital_Pathology_Transfer_Learning

## Background

Prostate cancer (PCa), the fourth most common cancer worldwide, is the sixth leading cause of cancer death among men, with 1.2 million new cases and more than 350,000 deaths in the world in 2018 [1]. PCa also has the second-highest incidence of all cancers in men [1]. The gold standard for the diagnosis of PCa is the visual inspection of tissue samples from needle biopsies or prostatectomies. The Gleason score (GS) is the standard grading system currently used to describe the aggressiveness of PCa [2].

The Gleason scoring assigns a number given the architectural patterns shown in prostate tissue samples observed under a microscope. The system describes tumor appearance and aberrations in the prostate tissue glands in biopsies or radical prostatectomy specimens. GS staging is required for treatment planning, and pathologists use it to help in prognosis and guide the therapy. The Gleason score comprises the sum of the two patterns (Gleason patterns, from 1 to 5) most frequently present in the tissue slide producing a final score in the range of 2 to 10. The revised Gleason score from the International Society of Urological Pathology (ISUP) is used in pathology routine [3].

Thanks to the recent improvements in digital pathology and the availability of slide scanners, the diagnosis is now also increasingly performed through the visual inspection of high-resolution scans of a tissue sample, known as Whole-Slide Images (WSIs) [4].

Computer assisted diagnosis (CAD) systems in digital pathology cover tasks such as the automatic classification or grading of a disease, segmentation of regions of interest like mitotic cells and tumor-infiltrating lymphocytes, as well as the retrieval of similar cases, among others [5, 6].

One of the aims of computational pathology (CP) is the development of CAD tools for helping pathologists in the analysis of WSIs, that can easily be over $100,000^2$ pixels in size. The lack of a large set of images annotated in detail (pixel level) is one of the constraints that researchers have when training deep learning models in computational pathology. Despite the lack of annotations, methods have shown partial success in medical imaging when trained with small sets of annotations using techniques such as multiple instance learning, active, transfer and weakly supervised learning [7–18].

Deep Convolutional Neural Network (CNN) models are currently the backbone of the state-of-the art methods to analyze prostate WSIs [7, 8, 17] in computational pathology. The success of CNNs relies on automatically learning the relevant features to classify the input images using a large set of annotated data with supervised learning. Obtaining big annotated datasets can be feasible for natural images, where the annotation effort is reduced by leveraging crowd-sourcing platforms such as Amazon mechanical turk [19]. In medical imaging, and particularly in histopathology, annotations from regions of interest require qualified personnel that undergoes years of training and often has limited time for such activities due to a heavy clinical workload. Manually increasing the number of annotations the number of annotations in a training dataset is well-known to improve the generalization performance in machine learning models. This is difficult in medical applications because of the costly annotations. For this reason, alternative sources of supervision using readily available and inexpensive labels (i.e., from clinical or pathology reports) are being studied to obtain larger annotated datasets without the time and cost of the pixel-wise annotations. *Transfer learning* refers to the set of techniques where models are trained on one dataset (or task) and then applied to another task (or dataset), where the features that are learned by the model can be reused. Empirical results show that it is better to transfer the weights of an ImageNet pre-trained network than to train from scratch for histopathology classification tasks[20]. The effect of transfer learning can be seen as a good choice of initial parameters for a deep neural network that will have a strong regularizing effect on its performance [21]. There are several motivations for transfer learning. One of the principal reasons is to discover generic features likely to be of interest for many classification tasks on similar data [22]. In the context of medical imaging, two types of transfer learning are commonly described in the literature [17, 23]: (1) where model features, for example the weights of a CNN architecture, are used to extract a continuous vector representation, i.e. a feature vector, from which a linear classifier is trained on top of this generic inferred representation to predict labels that were not used for the initial training of the model. (2) When a pre-trained model is used as the initialization for a second model and optimized or *fine-tuned* using a new set of labels that correspond to the new task. For computational pathology tasks, the second strategy of fine-tuning models from natural image datasets, such as ImageNet, was shown to yield better results than the off-the-shelf feature extraction [23]. Transfer learning is also a good strategy to speed-up model training time, since the models converge faster. *Strong labels* refer to manually delineated regions of interest in the images. These labels are also commonly called pixel-wise labels since usually each pixel can be assigned to one category depending on the contour of the manually delineated region. In computational pathology, such datasets with strong labels are rare because having a thoroughly annotated dataset is very expensive with such large images.

*Weak labels* refer to general categories such as cancer, benign tissue, or a score in a grading system of a specific organ, e.g., the Gleason grade in prostate cancer [7, 8] that are attached to an image as a whole and not to image regions. Spatial details are often lacking in pathology reports, since the exact location of the relevant areas is not given.

The line of research that investigates how to use inexpensive weak labels, known in the machine learning literature as *weakly supervised learning* has recently obtained good results in medical imaging, including computational pathology [8, 9, 15, 24–27]. In computational pathology, weak labels (or WSI-level labels), such as a grade for the entire image are often readily available for digital pathology applications since they are usually included in pathology reports summarizing the findings of the pathologist about the tissue slide or WSI. Weakly supervised learning allows to bypass the need for strong supervision by using weaker labels that are often easier to obtain with limited efforts, also in large quantities. However, this usually comes at the cost of requiring a large amount of weakly annotated data to reach good performance, i.e., a number of WSIs in the order of thousands [9].

A potential solution to the need for a large annotated dataset is to gather different supervision sources to train and test a model for a specific task leveraging from several datasets and transfer learning. Alleviating the potential of overfitting to one dataset allows obtaining a robust model that accounts for a higher variability of the sample classes [28].

This opens the question of how to use different types of supervision and data sources together in practice, since this can alleviate the requirement of many weak annotations. Furthermore, the trade-off between strong and weak supervision for CNN models in computational pathology is not clearly defined.

As discussed in the above paragraphs, weakly supervised CNN models pose feasible solutions to tackle classification tasks in computational pathology, particularly with the help of transfer learning approaches using the most frequently ImageNet pre-trained models. However, the computational pathology literature lacks methods dealing with the problem of building upon knowledge acquired in one histopathology dataset to another with similar characteristics or with the same underlying classification task.

There are many weakly annotated datasets available and also federated learning in hospitals is starting to be used [9]. The investigation of deep learning methods that leverage from different supervision levels, as well as knowing how many annotations are required for training such models, is of paramount importance for the practical deployment of computational pathology models in digital pathology laboratories.

In this paper fully and weakly supervised CNN models are compared using two openly accessible data sets and transfer learning, targeting prostate cancer scoring. Tissue microarrays with strong annotations and WSI with weak labels. We evaluated the performance gap between the models with an increasing number of strong annotations and the models trained with weakly labeled images and also both sources of data.

The organization of the article is as follows: first, we discuss the related work dealing with transfer learning and weakly supervised learning in computational pathology and how our contributions fit into the context of automatic PCa grading with deep learning. Then, in the experimental setup section, we describe the characteristics of the datasets, baseline CNN models and the transfer learning strategies. "Results" section, presents the results of the experiments and finally in "Discussion" section discusses the results in the context of similar approaches and models that might benefit from the strategies presented in the paper (Table 1).

**Table 1:** Number of TMA cores in the TMAZ dataset

Class/set	Train	Val	Test
Benign	61	42	12
GS6	165	35	88
GS7	58	25	38
GS8	120	15	91
GS9	26	2	3
GS10	78	14	13
Total	508	133	245

### Related work

The related work is summarized in Table 4. Both, the notion of combining weak and strong supervision [15] and transfer learning approaches [23] exist in the computational pathology literature. In this section we discuss the existing work and explain the contributions of this paper in the context of the related work. Recently, Otálora et al. [29] compared weakly supervised strategies for the fine-grained task of Gleason grading, reporting that the use of class-wise data augmentation and a DenseNet architecture using transfer learning lead to a kappa-score of $\kappa = 0.44$ in a set of 341 WSIs from the TCGA-PRAD repository. Nevertheless, the authors did not include strong supervision into the training of their models, despite having a limited number of WSIs. In the work of Ström [12] the authors obtained a $\kappa = 0.62$. The authors trained models with transfer learning using an ImageNet pre-trained InceptionV3 deep CNN architecture, for the Gleason scoring task using 1631 strongly-annotated WSIs. Arvaniti et al. [15] present a CNN architecture that combines weak and strong supervision for the classification tasks of high vs. low and Gleason scoring groups (6, $7=3+4$, $7=4+3$, 8, 9–10) used in clinical practice [30]. The model penalizes the weak supervision signal of patches by weighting them using predicted probability of the strong labels. The model using self-weighted weak supervision obtained an accuracy of 0.848 for the binary task and a Kendall’s $\tau$=0.540 for Gleason scoring using 447 WSIs and 886 annotated tissue microarrays.

Campanella et al. [9] use transfer learning and a massive dataset of more than 44,000 WSIs to weakly train CNN classifiers. The ImageNet pre-trained classifiers were trained using a multiple instance learning paradigm, using bags in which the assigned label referred only to a non-empty subset of elements in the bag, accounting for the inherent label noise. While their results paved the way for automated screening tools in computational pathology (where the pathologist can discard non-cancerous slides), their generalization to different clinical scenarios with highly heterogeneous data and fine-grained classes remains to be confirmed. Bulten [7] and colleagues use several CNN classifiers and immunohistochemistry labels to distill the noisy weak WSI-level labels obtaining a Gleason grade classifier with a performance comparable to the pathologist inter-rater agreement on a set of $\sim 1250$ IHC-H&E registered biopsies.

Recently, multiple instance learning techniques and attention models have had partial success in weakly supervised scenarios [9, 13, 26]. It was shown that despite the noise, the use of weak labels only allows a good performance for the low vs. high Gleason score classification [9, 31], as well as the Gleason score tasks [8]. This might be due to the definition of the Gleason scoring system itself, where the score directly correlates with the percentage of areas with the most frequently repeated Gleason patterns present in the image. However, to reach a clinical-grade performance, the number of images needed for training sometimes can be in the order of thousands of slides, as shown by Campanella et al. [9] for PCa detection.

### Contributions

This paper aims at contributing to answer two questions: (1) How to build up knowledge from different datasets to train histopathology image classifiers. We are particularly interested in how to distill the less-expensive, weakly annotated data in conjunction with few annotated regions. (2) How many annotations are necessary to train a model for automatic Gleason grading. For answering the questions we evaluate the trade-off of using small amounts of strong annotations from one data source, jointly with weak annotations from another data source to train supervised CNN models. CNN models are fine-tuned with different levels of pixel-wise labels of prostate cancer grading from TMA images. Then, the trained model is evaluated on Gleason scoring in the same type of TMA cores and then on WSIs from the TCGA-PRAD repository. Second, the pre-trained model is further fine-tuned with images that use the weak labels from the TCGA-PRAD reports to perform Gleason scoring at the WSI level. Despite the TMA dataset with strong labels having different visual characteristics from the WSI dataset with weak labels, fine-tuning the best model trained with TMA cores with the weak labels reduces the effect of domain shift, obtaining considerably better results than directly predicting on the external datasets and also better than training with the weak labels only, as reported in the results in "Results" section. In this paper we use only  400 slides, the weakly supervised training serves as a performance baseline to compare against other presented strategies. The main technical contributions of this paper are the following:

- A thorough evaluation of transfer-learning, using fixed amounts of strong labels, in the task of prostate cancer image classification with CNNs, using openly accessible datasets for evaluation of the generalization power of the model.
- Systematic evaluation of the CNN performance dependency on strong, weak labels and a combination of the two types of labels in the task of prostate cancer image classification with heterogeneous datasets.

## Methods

**Fig. 1:**
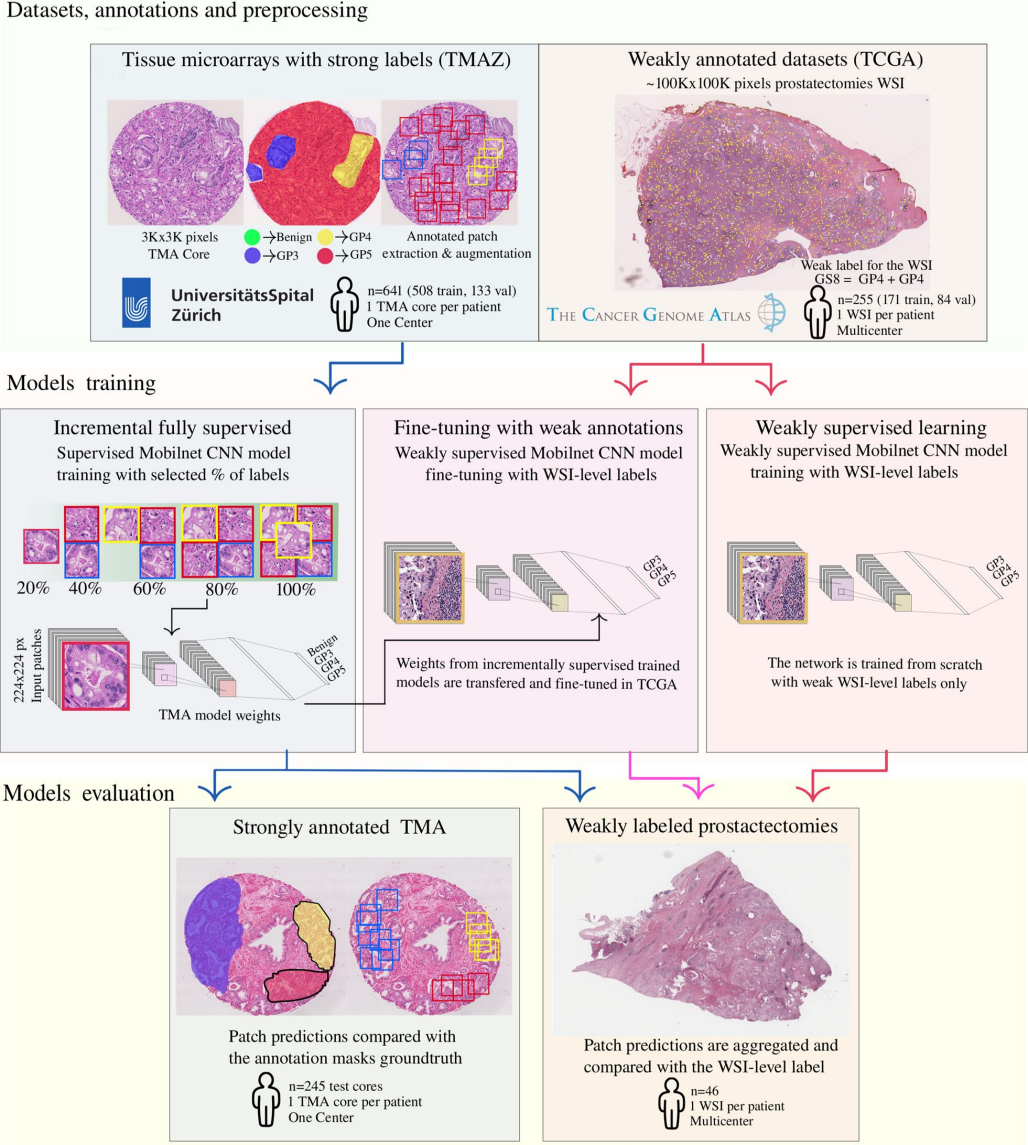
The main components of our approach: Datasets for PCa grading with strong and weak labels ("Datasets" section, CNN model training with three strategies "Datasets" section and third, the tests performed in two scenarios of PCa grading: tissue microarrays of prostate tissue and prostactectomy WSIs, "Results" section. The patches from the strongly labeled TMAs are used to train CNN models with an increasing number of annotations, evaluating the performance depending on the number of strong labels used for training. The ImageNet pre-trained models are either trained using only the weak WSI-level label or fine-tuned with WSI patches and weak labels, combining different sources of supervision. The models are tested in the two scenarios of PCa grading: tissue-microarrays, and prostactectomies. Arrows of the same color indicate the data or model input from the previous step

Figure 1 summarizes the overall workflow of the proposed approach to measure the dependency on annotations with different data sources in CNN models for PCa grading. First, we gather and preprocess the PCa grading datasets with heterogeneous characteristics ("Datasets" section), and with a different level of specificity in the labels. The first dataset consists of TMAs that are thoroughly annotated with pixel-wise Gleason pattern annotations. The second dataset is composed of WSIs of prostactectomies, in this case we used the WSI-level label that was extracted from the pathology reports.

Next, the CNN models are trained according to three strategies ("Datasets" section) as follows. The first one takes fixed percentages of pixel-wise annotations and trains fully supervised CNN models for each percentage. Like this we can evaluate how the performance of the CNN depends on the number of strong annotations provided for training the model. This is reported in "Results" section. The second strategy consists of taking each of the previously trained CNN architectures and fine-tune the weights using the weakly supervised dataset from the external source. Here, we evaluated if it was better to perform direct inference with the previously trained models or to transfer the weights of the fully supervised CNN and combining them using weakly supervised fine-tuning. In the third strategy, we trained the CNN model from scratch using only the WSI-level labels as supervision. While there are many non-relevant areas used for training the models, results show that the performance is robust in a test set with similar preparation conditions.

### Datasets

For the evaluation of the approaches, two openly accessible datasets are used in the experiments for Gleason scoring and Gleason pattern classification. The datasets originate from different sources. Despite the fact that the tissue is stained in all cases with hematoxylin and eosin (H&E) and that all the datasets are from prostate tissue, the visual appearance of the images shows many differences in the preparation. As shown in previous work [32, 33], not using color augmentation or stain-invariant methods decreases the performance of CNN models in WSI analysis. We account for this variability using image augmentations as described in "Datasets" section.

#### TMAZ: tissue microarrays with strong annotations

The first dataset is the pixel-wise pathologist-annotated tissue microarrays dataset released by Arvaniti et al. [34], referred to as TMAZ (Tissue MicroArrays Zürich). This dataset contains 886 prostate TMA cores, each of $3000^{2}$ pixels, from a cohort of 641 patients for model training/validation and 245 for testing, with one image core per patient. The TMAs were scanned at a 40x resolution (0.23 microns per pixel) at the University Hospital of Zurich (NanoZoomer-XR Digital slide scanner, Hamamatsu). Tumor stages and Gleason scores were assigned according to the criteria of the Union for International Cancer Control and the World Health Organization/International Society of Urological Pathology. In this dataset the annotated classes are benign epithelium, Gleason patterns 3, 4 and 5. Given the pixel-level region annotations, the Gleason score is computed using the two most prominent patterns present in the core, if only one pattern is present in the core, the Gleason score is twice that pattern. Overlapping *patches* corresponding to areas of $750^{2}$ pixels that cover enough gland context are extracted from the annotations mask. All the patches are scaled to $224^{2}$ pixels for feeding them to the CNN models, which are pre-trained with ImageNet [18]. The number of patches extracted from the annotations for each Gleason pattern are listed in Table 2 and the number of TMA cores used in the experiments is listed in Table 1.

**Table 2:** Number of patches for each Gleason pattern in the TMAZ dataset

Class/Set	Train	Val	Test
Benign	1831	1260	127
GP3	5992	1352	1602
GP4	4472	831	2121
GP5	2766	457	387
Total	15,061	3901	4237

Due to high class-imbalance, particularly for the high GP 5 and the benign tissue, class-wise data augmentation was applied

#### TCGA-PRAD: Prostactectomies with weak labels

The second dataset consists of 301 cases of prostatectomy WSIs from the public resource of The Cancer Genome Atlas (TCGA) repository of prostate adenocarcinoma (TCGA-PRAD) [35, 36]. Removal of the full prostate (radical prostatectomies) is only done in severe cases. There are thus no fully benign tissue slides in the TCGA-PRAD dataset. Having a benign prostatectomy implies a resection of the whole prostate when the tissue was healthy. When more benign lesions are present, the imaging technique chosen is usually an ultrasound or magnetic resonance imaging. In contrast with TMA, a single TMA core might contain only benign tissue due to its small size ( 0.7 mm in diameter vs. 3 cm of height in a prostatectomy slide) and the potential tissue sampling strategies, which do not always account for the tumor area.

From the 301 cases, 171 are used for training, 84 for validation and 46 for testing. The selected cases are a subset of all the available cases in TCGA-PRAD. We selected only the Formalin-Fixed Paraffin-Embedded slides. The other available slides also contained frozen sections, which include morphological changes due to the dehydration process that can lead to more noisy region extraction. Each WSI is paired with its corresponding primary and secondary Gleason pattern (weak) labels from the available pathology reports [35, 37]. Due to the massive size in pixels of a WSI, which can be over $100,000^{2}$ pixels, the patch extraction used for the supervised CNN training is performed only within relevant tissue regions. The HistoQC tool [38] is used to avoid extracting patches from connective tissue, also avoiding background and pen marks. After HistoQC generates a mask with the usable tissue areas, relevant areas (patches) need to be extracted. The blue-ratio ranking (BR), as described in Rousson et al. [39], is applied to obtain patches with high-cell density. We extracted 3000 patches of $750^{2}$ pixels at random locations within the HistoQC mask and selected the top 1000 blue-ratio ranked regions. To keep a comparable pixel size in the areas extracted and in the TMA images, all the patches are computed at a roughly 40$\times$ apparent magnification (0.25 microns per pixel) but include some heterogeneity. Each region is then down-sampled to a patch of $224^{2}$ pixels, which covers enough context and gland content. The detailed number of cases is given in Table 3.

**Table 3:** Number of WSIs from the TCGA-PRAD dataset that were used

Class/set	Training	Validation	Test
GS6	13	20	5
GS7 (3+4)	42	10	6
GS7 (4+3)	30	14	11
GS8	37	12	13
GS9-10	49	28	11
Total	171	84	46

### Model training

In the second row of Fig. 1, an overview of the approaches for the CNN model training is illustrated. The three strategies differ in what type of dataset is used (weakly labeled, strongly labeled, or a combination of both) and how the model is trained (incrementally fully supervised, fine-tuned with weak annotations, or only with weakly supervised learning). The architecture chosen for all the CNN models is the Mobilenet architecture [40], with a width Multiplier $\alpha =1$. Mobilenet was chosen as it allows comparability to the previously reported results of Arvaniti et al [14]. Mobilenet is a lightweight CNN architecture with fewer than $\sim$5 million parameters and was shown to obtain a performance that is comparable to pathologists on the TMAZ dataset [14]. Training the CNN classifiers from scratch is not optimal considering the relatively small size of the training set and the heterogeneity of data in color and tissue structures. For this reason, the models are initialized with pre-trained weights that were computed on the ImageNet challenge and then fine-tuned with the respective datasets [18, 41]. The ImageNet pre-trained Mobilenet architecture is kept constant throughout the three strategies. Dropout and weight regularization techniques were applied to avoid over-fitting. A dropout layer is placed in between the dense layers, with a probability of 0.2. This value was selected in line with the analysis made by Arvaniti and colleagues, where it is also stated that networks that were not regularized exhibit divergence in the validation cross-entropy loss scores. In the intermediate layers of the CNN, $L_2$ regularization was used, with a lambda parameter equal to 0.01. All the CNN models are trained to predict the Gleason pattern of an input image patch. In the case where strong labels are available, i.e. the TMAZ dataset, the performance is compared against the ground truth labels. For the weakly annotated TCGA-PRAD dataset, the models are optimized to predict the primary Gleason pattern reported for the WSI, and the Gleason score is computed using a majority voting of probabilities for the patches extracted in each WSI. This aggregation is simple but it reflects the nature of the grading system: adding the two most common patterns seen in the tissue sample. In the rest of this section, we discuss the details of each of the strategies.

To avoid model overfitting, it is usually good to have more samples that exploit the symmetries in histopathology images (where orientation does not influence the prediction), as well as to account for the subtle differences in stain and preparation methods. For this reason a pipeline for data augmentation is implemented for all the models using the Augmentor open-source library [42]. Augmentation is preferred over stain normalization since recent studies show the former has often better results than normalization techniques to tackle color heterogeneity [33, 43] Class-wise data augmentation is applied to alleviate the imbalance of the classes. The number of augmentations applied are inversely-proportional to the number of samples in each class as previous studies suggests [8]. The procedure includes four kinds of operations: rotation, flipping, morphological distortions and color augmentation. We obtain the equivalent of an extra half of training data by applying the operations to the images in the training set with a probability of 0.5. Rotation augmentation is performed by choosing a rigid rotation ($90^{\circ }$, $180^{\circ }$, or $270^{\circ }$) randomly with equal probability for each rotation. Flipping augmentation can be a vertical or horizontal flipping of the image. Morphological augmentation is a random elastic distortion applied to the image patch. The patch was divided into a 5*x*5 grid and the magnitude of the distortion was set to 8. Color augmentation was also applied, changing the colors of the input patches. The StainTools library [44] is used here, with the Vahadane stain extractor [45] and both sigma parameters set to 0.3. All the CNN architectures are optimized and evaluated using the internal validation partition and then evaluated in different test set scenarios as described in the results section.

#### Incremental supervised learning

In the supervised strategy, a comparison of the classification performance of patch-wise Gleason pattern classifiers is performed training CNN classifiers using an increasing number of strong annotations. The MobileNet architecture is fine-tuned using $750^{2}$ patches extracted from the pathologist annotations, following the approach of Arvaniti et al. [34]. The CNN is trained to classify the patches into the four patterns: benign tissue, Gleason pattern 3, 4 and 5. To evaluate the contribution of the number of annotations (strong supervision) to the performance of the models, five subsets of the TMA training set are extracted. Each subset has a percentage of samples and associated pixel-level annotations from the original training set: $20\% \subset 40\% \subset 60\% \subset 80\% \subset 100\%$. The number of patches in each subset are 3090, 6030, 9029, 12059 and 15061 patches, respectively. Following the original dataset partitions from the setup of Arvaniti et al. [46] the TMA training dataset includes cores from three arrays (ZT111, ZT199, ZT204), the validation set includes the cores from the ZT76 array and the test partition includes cores from the array ZT80. The TMA cores are heterogeneous in color representation, as it can be noticed by visual inspection of the arrays that are characterized by slight different stain colors. A fixed number of cores from each array from the training set are selected to have a balanced number of samples for each class. 30 patches are extracted from each TMA core. This value was chosen considering the size of each patch and the amount of tissue covered. It represents a good trade-off between the amount of overlap of the patches and the percentage of tissue extracted in the TMA, evaluated qualitatively. The patches are randomly generated within the annotation mask of the pathologist. We discard the patches with less than $60\%$ tissue. This criterion is applied for avoiding the extraction of uninformative patches from the slide background. To have more robust performance estimates we did not fixed the random seed in the experiments. We performed ten experiments to account for the different random seeds and SGD convergent solutions. The ten classifiers for each percentage were trained with the same configuration of hyper-parameters. All the models were trained for 25 epochs until convergence in validation performance was observed. The input for the models is a batch size of 32 image patches, using the Adam optimizer with a learning rate of 0.001 and a decay rate of $10^{-6}$, that are the standard values for many image classification tasks [47]. The learning rate was explored with the values of the set $\{10^{-5},10^{-4},10^{-3}, 10^{-2}, 10^{-1}\}$, and despite having robust performance measures regarding the learning rate, we observed the model to have a slightly better validation error with the value of $10^{-3}$.

#### Fine-tuning with weak annotations

Fine-tuning a model that is initially trained with few annotated samples using a new set of samples for the same underlying task (i.e., Gleason grading) is a suitable technique for combining several levels of supervision since it allows to reuse the knowledge acquired on one dataset to others where the input distribution might shift slightly, for example due to slide preparation. In this strategy, we start with an ImageNet pre-trained Mobilenet CNN, which is initially fine-tuned with a fixed number of strongly annotated data from the TMAZ dataset. Finally, the model is further fine-tuned with the regions extracted from the whole slide images of the TCGA-PRAD dataset. Strictly, this means that there are two stages of fine-tuning, the first one from the ImageNet pre-trained models to the TMAZ and then from TMAZ to TCGA dataset.

The loss function of the classification models minimizes the categorical cross-entropy between the predicted and the pathologist-reported primary Gleason pattern of each of the WSIs. The models are trained at the patch level and then the results are aggregated using majority voting to obtain the Gleason score. Since there are ten models for each percentage of supervised annotations with the fully supervised training, each model is fine-tuned and the performance for each percentage of strongly annotated data is averaged over the ten models.

The transfer learning models are trained with the same hyperparameters as the supervised models. They are trained for 5 epochs, with a batch size of 32 samples, using the Adam optimizer with a learning rate of 0.001 and a decay rate of $10^{-6}$. As in the TMA, also the patches from the WSIs are highly heterogeneous in color representation. In total, there are 50 fine-tuned Mobilenet models using the TCGA-PRAD dataset: There are 10 models for each annotation percentage.

#### Weakly supervised learning

In this strategy, we use only the weakly labeled dataset to train the network to predict the global Gleason score. Training a model only with weak labels is a challenging scenario since many of the patches that are fed to the network might not contain the relevant visual characteristics or patterns that are associated with the global Gleason score [8, 48].

The weakly supervised models are trained with all 100% of the weak labels available. The models are trained for 5 epochs, with an input batch size of 32 samples and the Adam optimizer with a learning rate of 0.001 and a decay rate of $10^{-6}$. Since the prediction is at the patch level but the labels used for evaluation are at the WSI level, the predictions have to be aggregated. The WSI label computed by taking the majority voting of the most frequently predicted Gleason patterns. Similar to the training phase, there are two test sets for evaluating the models of the three proposed strategies: the strongly annotated TMAZ weakly annotated TCGA-PRAD test sets. The classification performance is measured as the inter-rater agreement with the ground truth. The raters can be either the pathologist who annotated the dataset and made a report, or the prediction model, that assigns classes to the image patches. The used performance measure is Cohen’s kappa ($\kappa$) that is often used in PCa grading [12, 14, 15] because it really chose the agreement between the algorithm and the human raters and is also used to quantify inter-rater disagreement. A perfect agreement has a score of $\kappa = 1$ and since $\kappa$ is normalized by random chance, a random assignment of ratings (classes) has a $\kappa = 0$. The *kappa* score that is used throughout reporting all the results in this section is the quadratically weighted $\kappa$ that penalizes predicted values that are far from their actual class, i.e. if the annotation for a patch is GP4 and the predicted class is GP5, it is penalized less than if the predicted class is benign. The quadratically weighted $\kappa$ score is defined as:

$$\begin{aligned} \kappa = 1 - \frac{\sum _{i,j}w_{i,j}O_{i,j}}{\sum _{i,j}w_{i,j}E_{i,j}}, w_{i,j} = \frac{(i-j)^{2}}{(N-1)^{2}} \end{aligned}$$

where *i*, *j* are the ordered scores, $N=5$ is the total number of Gleason scores, or $N=4$ in the case of Gleason patterns. $O_{i,j}$, is the number of images that were classified with a score *i* by the first rater and *j* by the second. $E_{i,j}$ denotes the expected number of images receiving rating *i* by the first expert and rating *j* by the second. The quadratic term $w_{i,j}$ penalizes the rating-predictions that are separated. When the predicted Gleason score is far from the ground-truth class, $w_{i,j}$ gets closer to 1.

## Results

After all models are trained, an evaluation in different PCa grading scenarios is performed. In order to evaluate the generalization power of the models, they are evaluated on different test-sets where possible. Results suggest that the strategy of training with a small number of annotations and fine-tuning the models with weak labels of the data at hand, is better than performing direct inference with pre-trained models and than training the models from scratch using the weak labels.

The plots presented in this section have 5 data points (except for the weakly supervised training), corresponding to the subsets of strong annotations used to train the models (20%, 40%, 60%, 80%, 100%). Each of the five points represents the average of the results of the ten trained models for each percentage. Along with the average, confidence intervals at 95% are drawn as a shaded area. Between each pair of points, the intermediate values are interpolated to display the performance tendencies. The figures and tables display the performance on the different test partitions that are used neither for training nor model selection. The tests carried out for each strategy are described in the following subsections (Table 4).

**Table 4:** Reported performance for prostate cancer grading and scoring using deep learning models

Reference	Classes	Results	#Patients	Annotations	Multicenter
Arvaniti [14]	GS6,GS7,GS8,GS9,GS10	$\kappa = 0.75$	641	Strong	No
Nagpal [10]	GS6,GS7,GS8,GS9,GS10	ACC$= 0.70$	342	Strong	Yes
Burlutskiy [11]	With/out basal cells	$F_{1} = 0.80$	229	Strong	No
Ström [12]	ISUP: 1,2,3,4,5	$\kappa = 0.67$	976	Strong	Yes
Otálora [8]	GS6, GS7, GS8, GS9, GS10	$\kappa = 0.44$	341	Weak	Yes
This work	ISUP: 1,2,3,4,5	$\kappa = 0.52$	341 WSI + 641 TMA	Weak and strong	Yes
Arvaniti [15]	ISUP: 1,2,3,4,5	$\tau = 0.54$	447 WSI + 641 TMA	Weak and strong	Yes
Bulten [7]	ISUP: 1,2,3,4,5	$\kappa = 0.72$	1243	Weak and strong	Yes
Campanella [9]	Benign versus cancer	AUCs of 0.98	7159	Weak	Yes

The first four rows correspond to strongly supervised methods using pixel-level annotations. The last four rows are weakly supervised methods that use global labels. Multi-Center studies involve training with images from multiple institutions, which increases complexity and requires good generalization performance

### Incremental supervised learning (TMAZ test set)

In this setup the MobileNet classifiers, trained with an increasing number of annotations from the TMAZ dataset, are used to perform inference on the TMAZ test set. The full training and test procedures on the TMAZ dataset correspond to the leftmost branch (blue arrows) in Fig. 1. On the TMAZ test set, the evaluation is straightforward: for each percentage of annotation, the best model for each of the ten repetitions, as evaluated on the validation partition, is selected. Then, the ten models are used to predict the Gleason patterns and aggregate them to compute the Gleason score for the patches in each TMA core. In this evaluation the patches are of the same center of the input patches and the labels are the same that were used for training of the models.

**Fig. 2:**
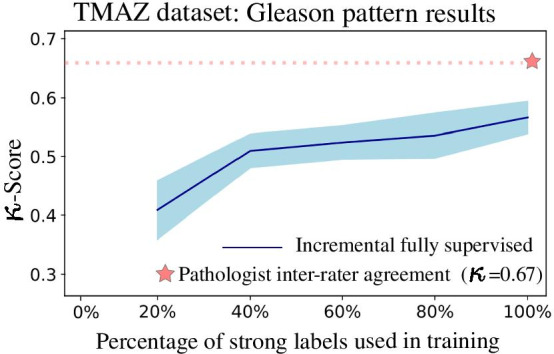
Results for the average performance of the trained models as measured by $\kappa$-score as function of the strong annotation percentage used for training

In the case of Gleason pattern classification on the TMAs using the models trained with strong labels, the performance is monotonically increasing, as shown in Fig. 2 .

The average performance of the models when using 100% of the annotations is $\kappa = 0.55 \pm 0.03$, which is comparable to the performance reported by Arvaniti et al. [14] of $\kappa = 0.55$.

**Fig. 3:**
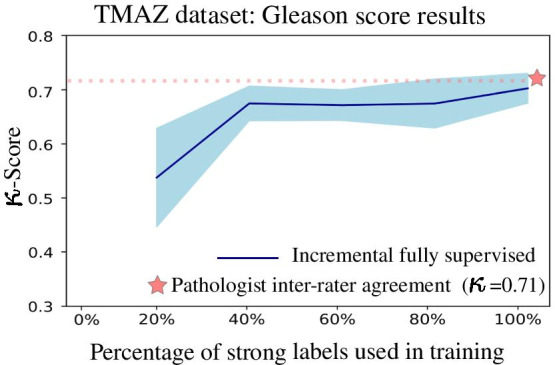
Results for the average performance of the trained models as measured by the $\kappa$-score as function of the strong annotation percentage used for training

The plot in Fig. 3 represents the Gleason score classification on TMAs using the models trained with strong labels. As reference, the inter-pathologist agreement is represented in both cases with a star. The model performance increases until 40% of the annotations are used and then it remains approximately stable until 100% of the annotations are used. The average performance of the 10 models when using 100% of the annotations is $0.69 \pm 0.02$, which is comparable to the pathologists agreement of $\kappa = 0.71$.

### Weakly labeled prostatectomies (TCGA-PRAD test set)

**Fig. 4:**
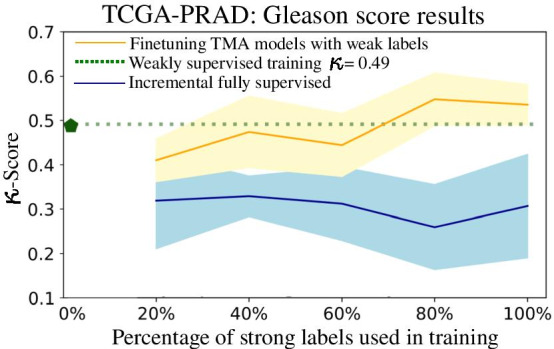
Results for the average performance of the trained models using the TCGA-PRAD test dataset. The performance is measured by the $\kappa$-score as function of the strong annotation percentage

The results of the three strategies on the TCGA-PRAD evaluation are shown in Fig. 4 and in Table 5 the results are summarized for the case where the inference and fine-tuning models use 100% of the strong labels. To compare the weak ground-truth label with the prediction of the models, the individual patch predictions need to be aggregated. The probabilities inferred for all the 1000 patches of each WSI are aggregated with a majority voting rule. The following rule is applied to take into account WSIs with the same primary and secondary Gleason patterns: if the most frequently represented pattern has more than twice the number of patches of the second, then this pattern is assumed to be both the primary and the secondary Gleason pattern. In the prostatectomies TCGA-PRAD test set, the evaluations correspond to the three arrows in the weakly labeled prostactectomies branch of Fig. 1. where all of the three model training strategies are evaluated:

1. *Incremental fully supervised learning models* In this test, the MobileNet classifiers trained with an increasing number of annotations from the TMAZ dataset, are used to perform inference on the patches of the TCGA-PRAD whole slide test images. Here, the models perform inference in the automatically extracted region patches, without further fine-tuning. The hypothesis is that the model can be transferred, since the patterns learnt on the TMAZ dataset are similar to those using the TCGA-PRAD WSIs (which is likely the case since the grading system is the same and pathologists usually do not have problems in grading in either of the image sources). Despite the difference in image size and visual characteristics, the trained models should learn high-level representations of the visual Gleason patterns that are transferable to external prostate cancer image datasets.
2. *Weakly supervised training* This is the case where the models do not use the annotations from the TMA dataset at all, but only the weak labels as the source of supervision. The last dense layer of the model predicts both the primary/secondary Gleason pattern. In this case, the model should capture the relevant patterns on the TCGA-PRAD dataset due to the large number of selected patches used to train ($\sim$171,000 patches), which are also processed with the data-augmentation pipeline described in "Datasets" section.
3. *Fine-tuning TMA models with weak labels* These models infer using the fine-tuned TCGA-PRAD features. In this case the transferred features are learned on the TMAZ dataset. The model weights used in each percentage as initialization are the weights of the best TMA model (as measured by $\kappa$-score in the TMAZ validation set) for the corresponding percentage of the annotations, changing the last dense layer to predict both the first and second Gleason patterns. In this case, the model is expected to further reduce the difference between the datasets by adapting with the particularities of the TCGA-PRAD dataset.

Figure 4 shows the performance for the three strategies on the TCGA-PRAD test set. The performance is measured by the weighted Cohen Kappa ($\kappa$-score) as a function of the percentage of TMAZ annotations used to train the models.

**Fig. 5:**
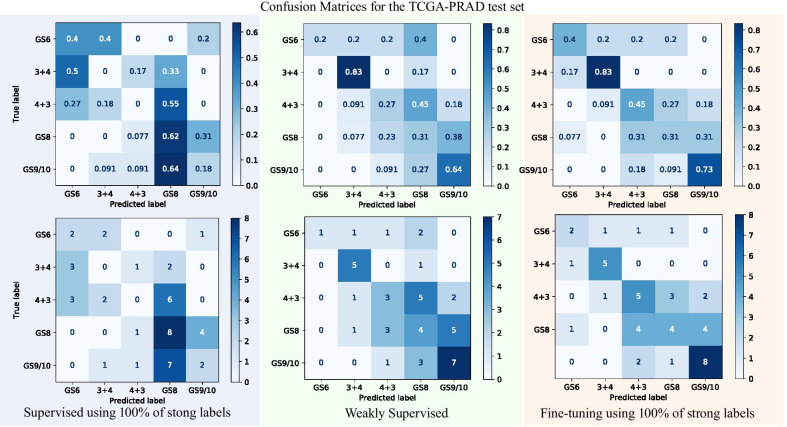
Confusion matrices displaying the correct cases classified for each of the five ISUP grades (diagonals of the matrices) in the TCGA-PRAD dataset. The top row shows the normalized matrices from the bottom row matrices displaying the total number of cases

The performance of the first strategy of the models using incremental full supervision is shown as the blue line. This strategy reaches $\kappa =0.31 \pm 0.13$ as the average for the 10 trained models when using the 100% of the annotations. This strategy obtains the worst performance, regardless of the number of the annotations used. The second strategy of weakly supervised training, i.e. fine-tuning Mobilenet from ImageNet weights using only the TCGA-PRAD dataset, achieves $\kappa =0.49\pm 0.08$ and is represented with the green line. The weakly supervised strategy obtained better performance than the incremental fully supervised models, despite being trained only with the noisy, weak labels. The third transfer learning technique that fine-tunes the incremental fully supervised models reaches a $\kappa =0.52 \pm 0.05$ as performance when fine-tuning the TMAZ model with 100% of the annotations. The performance for the fully supervised models with each percentage of data used is represented as the orange line. This transfer learning strategy outperformed the other two strategies and it suggests a performance increase when the number of annotations used to train the fine-tuned models also increases. The performance seems to be more robust, i.e., having a narrower confidence interval, as long as more annotations are provided to the fine-tuned models. The performance gap between the incremental fully supervised strategy is broader than between the weakly supervised strategy. The results for Gleason score and primary and secondary Gleason patterns are summarized in Table 5. The correct and misclassified cases for each ISUP grade and each method are in the confusion matrices shown in Fig. 5.

**Table 5:** Performance for the evaluated methods on the test data of the TCGA-PRAD dataset

Model	$\kappa$-GS	$\kappa$-PGP	$\kappa$-SGP	Avg. acc.	Error rate	Micro-precision
Supervised (100%)	0.30 ± 0.13	0.23 ± 0.16	0.10 ± 0.1	0.51 ± 0.06	2.45 ± 0.33	0.27 ± 0.05
Weakly supervised	0.49 ± 0.08	0.36 ± 0.11	0.30 ±0.09	0.67 ± 0.03	1.65 ± 0.18	0.43 ± 0.04
Fine-tuning (100%)	0.52 ± 0.05	0.34 ±0.10	0.40 ± 0.10	0.69 ±0.02	1.51 ± 0.14	0.46 ± 0.03

## Discussion

In the following paragraphs we discuss the results and trade-offs in sample efficiency of each of the methods. An increase of the classification performance on the TMA dataset is observed when increasing the number of annotations used to train the model. This was expected because of the controlled conditions of the experiment and annotations, where all the data originates from the same pathology laboratory. The performance trend suggests that more pixel-wise annotations can further increase the performance in Gleason pattern classification. The performance for Gleason scoring is always above the performance of Gleason pattern classification. This may be due to the way in which the Gleason score is calculated, since the order of the patterns is irrelevant to the final score in this evaluation, thus making the task easier.

The inter-dataset heterogeneity is evident when the best model trained with the TMA dataset failed to generalize on the TCGA-PRAD dataset directly. The image heterogeneity in the TMAZ dataset is lower than in the TCGA-PRAD dataset. Furthermore, the strongly annotated training dataset is relatively small (up to 15,000 patchs). Besides the visual differences, the lack of annotations makes the patch extraction process more prone to errors, which is tackled only partially by extracting patches from usable tissue masks only and ranking the patches with the blue-ratio criterion. The augmentations used for the TCGA-PRAD dataset make the training of the CNN more robust to tissue appearance.

For this, the fine-tuning alleviated the performance decrease considerably, surpassing the performance of the weak training only, which shows that the model is correctly leveraging upon the TMA weights transferred and adjusting to the particularities of the TCGA-PRAD dataset.

There is an important gap between the performance of the model in the controlled TMAZ dataset with $\kappa = 0.69 \pm 0.02$, and the best models on TCGA-PRAD $\kappa = 0.52 \pm 0.05$ which suggests that there is room for improvement by designing a model that can better leverage the usage of a combination of a small set of strong labels and a large set of weakly annotated data. The most effective transfer learning method for the WSI dataset was fine-tuning the trained TMA model weights using the weakly annotated patches, despite the fact that these patches are less reliable than those from the TMAZ dataset (because they refer to the global WSI diagnosis rather than local structures). This shows a similar behaviour to what occurs in transfer learning with CNN models trained on natural images [41], where even if the task or dataset differs substantially there is still a set of basic features that linger to the fine-tuned models and allow to have better generalization.

Our strategy is easy to implement and applicable to different scenarios but also has a few limitations. The principal limitation is that weak labels introduce some degree of noise that is difficult to quantify. There are no ground-truth regions of interest to compare with; nevertheless, this problem is evident in the performance gap from strong supervision to the weakly trained models. For the limitation introduced by the noise in the weak labels, we foresee improvements by adopting attention mechanisms and multiple instance learning models that allow the model to discard the noisy non-relevant samples and already proved useful and effective in histopathology use cases [9, 13, 26].

Second, the combination of weakly and fully supervised learning are the best experimental results at the whole slide image level, consistent with previous similar work using such a combination [15]. An interesting observation is that for achieving the best performance, it comes with the cost of using at least the 70% of the strong annotations in conjunction with all the weak labels to outperform the baseline of weakly supervised training. The need for such a large number of strong labels might be a drawback in the case of having very few strong labels. This need for additional supervision can be reduced using active and semi-supervised learning techniques [48, 49]. We also performed internal experiments using a large set of prostate biopsies(https://www.kaggle.com/c/prostate-cancer-grade-assessment/, dataset is currently under embargo). The results also showed the same trend and similar results as for the TCGA dataset, thus making the approach applicable to the commonly used images in clinical practice.

## Conclusion

In this paper, transfer learning strategies that combine strong and weak supervision are evaluated in two cases of prostate cancer image classification in histopathology: tissue microarrays of prostate tissue and prostatectomy WSIs. The results of the techniques show that the Gleason pattern classification performance of a CNN model can be improved using a combination of strong and a large amount of weak labels using transfer learning.

The performance increases in the controlled TMA scenario with a larger number of annotations used to train the model. Nevertheless, the performance is hindered when the trained TMA model is applied directly to the more challenging WSI classification problem. This demonstrates that a good pretrained model for prostate cancer image classification may lead to the best downstream model if fine-tuned on the target dataset but it may not be the most transferable and generalizable pretrained model otherwise. In future work, we plan to close the generalization gap even further between the weakly supervised trained models and the fully supervised ones, by better leveraging on the combination of both sources of supervision, by designing and training better semi and weakly-supervised learning models.

## Data Availability

The results shown in this paper are partly based upon data generated by the TCGA Research Network: https://www.cancer.gov/tcga. The datasets described in this paper are publicly available. The TMAZ dataset can be found here: https://dataverse.harvard.edu/dataset.xhtml?persistentId=doi:10.7910/DVN/OCYCMP. The TCGA-PRAD whole slide images can be found here (Only diagnostic slides were used): https://portal.gdc.cancer.gov/projects/TCGA-PRAD The source code for reproducing the experiments will be available upon publication of the manuscript.
